# Dosimetric validation of Monaco treatment planning system on an Elekta VersaHD linear accelerator

**DOI:** 10.1002/acm2.12188

**Published:** 2017-09-25

**Authors:** Ganesh Narayanasamy, Daniel L Saenz, Dewayne Defoor, Niko Papanikolaou, Sotirios Stathakis

**Affiliations:** ^1^ Department of Radiation Oncology University of Texas Health San Antonio San Antonio TX USA; ^2^ Department of Radiation Oncology University of Arkansas for Medical Sciences Little Rock AR USA

**Keywords:** dosimetric validation, Monaco, Monte Carlo, treatment planning system, VersaHD

## Abstract

The purpose of this study is to perform dosimetric validation of Monaco treatment planning system version 5.1. The Elekta VersaHD linear accelerator with high dose rate flattening filter‐free photon modes and electron energies was used in this study. The dosimetric output of the new Agility head combined with the FFF photon modes warranted this investigation into the dosimetric accuracy prior to clinical usage. A model of the VersaHD linac was created in Monaco TPS by Elekta using commissioned beam data including percent depth dose curves, beam profiles, and output factors. A variety of 3D conformal fields were created in Monaco TPS on a combined Plastic water/Styrofoam phantom and validated against measurements with a calibrated ion chamber. Some of the parameters varied including source to surface distance, field size, wedges, gantry angle, and depth for all photon and electron energies. In addition, a series of step and shoot IMRT, VMAT test plans, and patient plans on various anatomical sites were verified against measurements on a Delta^4^ diode array. The agreement in point dose measurements was within 2% for all photon and electron energies in the homogeneous phantom and within 3% for photon energies in the heterogeneous phantom. The mean ± SD gamma passing rates of IMRT test fields yielded 93.8 ± 4.7% based on 2% dose difference and 2 mm distance‐to‐agreement criteria. Eight previously treated IMRT patient plans were replanned in Monaco TPS and five measurements on each yielded an average gamma passing rate of 95% with 6.7% confidence limit based on 3%, 3 mm gamma criteria. This investigation on dosimetric validation ensures accuracy of modeling VersaHD linac in Monaco TPS thereby improving patient safety.

## 1 INTRODUCTION

Fraass et al had reported that a dose error of 5% could lead to a tumor control probability (TCP) change of 10–20% and an even higher change in normal tissue complication probability (NTCP).^1^ Noticeable clinical effects of dose errors of 7% have been reported in TG‐65 by Papanikolaou et al^2^ Reducing the uncertainties in dose calculation will reduce the overall uncertainty in delivered dose to within 5% as recommended by International Commission on Radiation Units and Measurements (ICRU) report 24.^3^

Monte Carlo (MC)‐based dose calculation engines have been reported to have the potential to better the 3% requirement for dose uncertainty.^4^ MC uses a stochastic method to calculate dose from first principles that accounts for material details of the treatment head.^5^ MC has been shown to calculate accurate dose distributions, especially in heterogeneous patient tissues involving complex electron transport trajectories.^6^ Its advantage over conventional dose engines is that the uncertainties are independent of setup leading to increased confidence in the calculated dose distribution. However, MC dose calculation engines suffer from increased planning time, statistical uncertainties from limited number of histories sampled, mismatch between measured and modeled data, and conversion of CT data to physical density data.^7^ This calls for validation of MC dose calculation with dosimetric measurements followed by clinical studies.^8^

In this study, we perform the dosimetric verification of Monaco TPS version 5.1 (Elekta CMS, Maryland Heights, MO, USA) on an Elekta VersaHD linac (Elekta, Crawley, England). This study was initiated to investigate the dosimetric accuracy prior to clinical usage due to two main reasons. First, the newly designed Agility head has a dynamic leaf guide with a variable thickness combined with the 160 multileaf collimators (MLC) without a backup jaw. The MLC‐defined collimation in one axis could lead to higher penumbra and alternate head leakage spectrum.^9^ Second, the high dose rate FFF beams have a lower out‐of‐field dose, different electron contamination spectra, and possibly lower mean energy.^10^, ^11^, ^12^

Calibrated ionization chamber‐based point dose measurements were followed by diode array‐based 2D dose measurements which were compared with planned dose distribution using Gamma analysis. Monaco calculated dose distributions in a few head and neck (H&N), brain, lung, and abdominal treatment sites were compared against dosimetric measurements. We have investigated overall performance of treatment delivery by quantifying the confidence limits on dosimetric accuracy using benchmarks set by task group 119 (TG‐119).^13^

## 2 METHODS AND MATERIALS

The treatment machine is an Elekta VersaHD linac with 6 MV, 10 MV, 18 MV, 6 MV flattening filter free (FFF), 10 MV FFF photon energies, as well as 6 MeV, 9 MeV, 12 MeV, and 15 MeV electron energies.

### 2.A Commissioning beam data

Monaco commissioning beam data acquisition was based on the manufacturer instructions as well as recommendations of AAPM TG‐106.^14^ A PTW MP3‐M water tank (PTW, Freiburg, Germany) was used with a PTW Semiflex 31010 chamber (0.125 cm^3^ active volume) or a PTW Diode P dosimeter (active volume = 0.03 mm^3^) for dosimetric measurements and data were processed using PTW's MEPHYSTO mc^2^ Navigation software. The percent depth doses (PDD), output factors, and beam profiles were acquired at 90 cm source‐to‐surface distance (SSD) for square field sizes from 1 × 1 cm^2^ up to 40 × 40 cm^2^. PDDs, output, and wedge factors were acquired with a PTW Diode P dosimeter (active volume = 0.03 mm^3^) for fields ≤5 × 5 cm^2^ and with the PTW Semiflex chamber for field sizes ≥5 × 5 cm^2^. A daisy chain approach was used in integration of data, and measurements of the two dosimeters were normalized to a 4 × 4 cm^2^ field size. All profile scans were performed using the PTW diode P dosimeter for better spatial resolution. This includes inplane, crossplane, and diagonal profile scans acquired at depths of *d*_max_, 5 cm, 10 cm, and 20 cm. Penumbra was measured from the spatial distance between the 80% and 20% of the central axis value in the profile scan of the flattened beam. For FFF beams, the penumbra normalization technique where‐in the profiles were normalized to the largest field size was utilized.^15^

### 2.B Dosimetric verification

**Figure 1:**
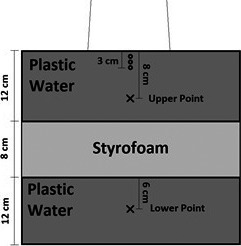
Schematic of phantom used for end‐to‐end testing of point dose measurement.

In this study, the virtual linac model built by Elekta was verified using a set of measurements recommended in AAPM's Medical Physics Practice Guideline (MPPG) report 5a.^16^ Point dose verification measurements were made on a plastic water (CNMC, Nashville, TN, India) phantom with overall dimensions of 30 × 30 × 32 cm^3^ with an 8 cm slab of Styrofoam (for heterogeneous medium) centered in the middle, as outlined in Fig. 1. The phantom was scanned on a GE LightSpeed 16‐slice CT scanner (GE Healthcare, Waukesha, WI, USA) at 2.5 mm slice spacing and exported to Monaco TPS. The density of the plastic water was overridden to 1.04 g/cm^3^, as mentioned in the manufacturer's guidelines. Two points of interest (POI) were added in the Monaco plan representing the location of the ionization chamber. The upper point was placed at 8 cm along the central axis and the lower point at 26 cm depth (6 cm behind the Styrofoam slab) for heterogeneity testing. Five fields were devised for testing dosimetric accuracy including: (a) an open 10 × 10 cm^2^ field, (b) 10 × 10 cm^2^ field with 30° wedge, (c) 20 × 20 cm^2^ field at 110 cm SSD, (d) 30 × 30 cm^2^ field at 20° oblique gantry incidence, and (e) a rectangular 20 × 5 cm^2^ field. The wedge field was not included for the FFF beam measurements. In the oblique field at 20° gantry angle of incidence, the upper measurement point was 2.5 cm off the central axis. In the rectangular 20 × 5 cm^2^ field, the jaws define the 20 cm edge. All the fields were measured at the upper point of measurement. In addition, the open 10 × 10 cm^2^ field was measured at the lower point of measurement. For the electron energies, two fields were devised with a 10 × 10 cm^2^ cone applicator for the phantom setup at (a) 100 cm SSD and (b) 105 cm extended SSD. The points of measurement were at a depth of 1, 2, 2, and 3 cm for the 6, 9, 12, and 15 MeV, respectively. Two‐hundred MU was prescribed for all the photon and electron fields. In addition, Monaco‐reported CT number electron density of various known materials was compared against known values. The CT images of CT electron density phantom (Gammex RMI, Middelton, WI, USA) were read into Monaco TPS and the density, mean Hounsfield units (HU) of various inserts were compared with expected values.

A calibrated PTW Semiflex 31013 ionization chamber (PTW, Freiburg, Germany) with an active volume of 0.3 cm^3^ was utilized for measurements along with a PTW Unidos webline electrometer. The values of *P*_ion_ and *P*_pol_ were measured during TG‐51 calibration as a part of commissioning.

### 2.C IMRT test fields

A set of eight static and IMRT fields were available to authenticate the MLC parameters in the photon MC model. Two open fields 10 × 10 cm^2^ and 20 × 20 cm^2^ indirectly verify the absolute dose calibration and field flatness, symmetry, respectively. The “3ABUT” field with three abutting field segments and “7segA” field consisting of seven segments were used to validate MLC leaf tip offset position. The “FourL” field made of four L‐shaped segments was used to adjust the MLC transmission. The dynamic MLC field “DMLC” was used to authenticate the combination of MLC transmission, leaf offset, and leaf tip leakage. The high‐density MLC field “HDMLC” and high‐dose IMRT field “HIMRT” were representative clinical fields that fulfill the purpose of a final endorsement of the adjustments made using the previous fields to ensure that the plan agreement is appropriate.

IMRT test fields optimized in Monaco TPS were subjected to dosimetric testing by comparison of planned dose distribution against measured data using gamma analysis. The fields were measured for all the photon energies on the Delta^4^ bi‐planar diode array (ScandiDos, Uppsala, Sweden). The array consisting of 1069 diode dosimeters with 5 and 10 mm respective spacing in the center and peripheral region was specifically commissioned for the VersaHD linac, as specified in the manufacturer's guidelines. Gamma analysis was performed using a 2% dose deviation (DD), 2 mm distance‐to‐agreement (DTA) criteria, and 10% dose threshold based on a global normalization.

### 2.D Patient data validation

We used the VersaHD virtual machine model in Monaco TPS to calculate dose for H&N, brain, lung, and pelvis treatment sites which were compared against measurements made on the Delta^4^ diode array. The results of the gamma analysis were tabulated for 3% DD, 3 mm DTA, and 10% dose threshold based on a global normalization. As proposed by Palta et al, a quantified degree of agreement that should be acceptable based on the “confidence limit” (CL) was utilized here.^17^ Based on the modified definitions laid out in TG‐119, the CL is the reduction from 100% of points passing the gamma criteria summed with 1.96 times the standard deviation (SD). It is expected that 95% of measurements would fall within this CL based on normal distribution. The measurements were performed five times for each of the eight patient plans.

## 3 RESULTS

### 3.A Commissioned beam data

**Figure 2:**
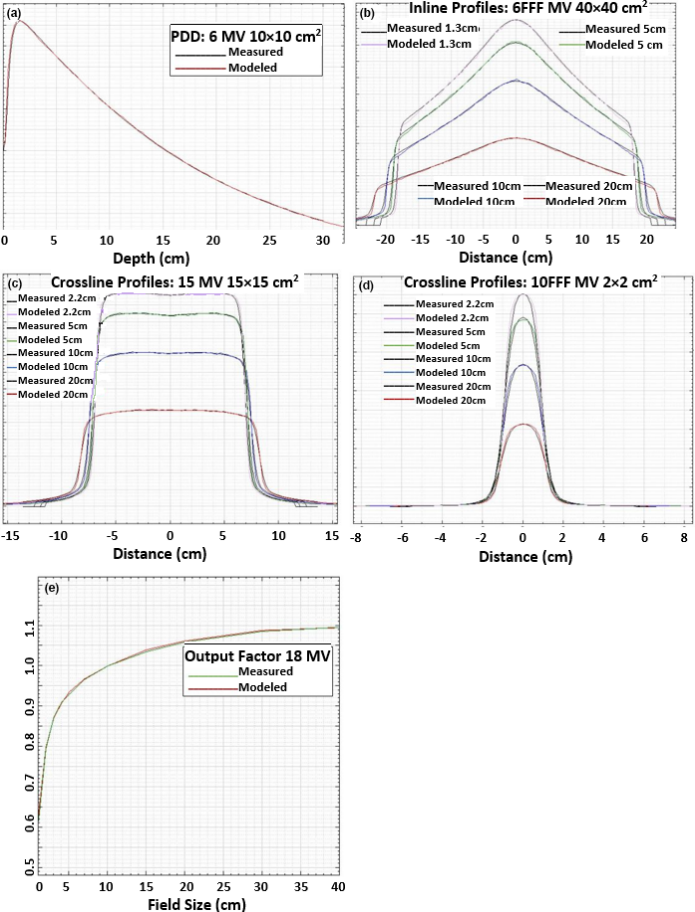
Comparison between measured and modeled VersaHD beam data: (a) PDD of a 6 MV 10 × 10 cm^2^ field; (b) inline profiles of a 6 MV FFF 40 × 40 cm^2^ field measured at depths of 1.3, 5, 10, and 20 cm; (c) crossline profiles of a 10 MV 15 × 15 cm^2^ field measured at depths of 2.2, 5, 10, and 20 cm; (d) crossline profiles of a 10 MV FFF 2 × 2 cm^2^ field measured at depths of 2.2, 5, 10, and 20 cm; and (e) output factor of a 18 MV photon beam, respectively.

The virtual machine was modeled based on the commissioning beam data and a comparison between some of the measured and modeled beam data was illustrated in Fig. 2. Shown in Figs. 2(a)–2(e) were PDD of a 6 MV 10 × 10 cm^2^ field, inline profiles of a 6 MV FFF 40 × 40 cm^2^ field, crossline profiles of a 10 MV 15 × 15 cm^2^ field, crossline profiles of a 10 MV FFF 2 × 2 cm^2^ field, and output factor of a 18 MV photon beam, respectively.

The difference between modeled and measured 80–20% penumbra values for an inplane scan of a 10 × 10 cm^2^ field was 1.2, 0.6, 1.1, 0.8, and 1.0 mm, respectively, for 6, 6 FFF, 10, 10 FFF, and 18 MV photon beams.

### 3.B Dosimetric verification

The parameters of the fields and the point dose differences are tabulated in Table 1 for all photon energies. Agreement in the measured data was within 2% and 3% in homogeneous and heterogeneous phantom for photon energies, respectively. The agreement in the measured data was within 2% for the electron energies in homogeneous phantom, as tabulated in Table 2. These results agree with the recommended tolerances mentioned in MPPG report 5a. The density and mean HU values of various inserts in a CT electron density phantom were tabulated in Table 3. Also shown are the comparable HU values from the virtual CT simulator, GE LightSpeed 16‐slice CT (GE Healthcare, Waukesha, WI, USA).

**Table 1:** Point dose differences between measurements and Monaco TPS calculated data in the Plastic water at upper point (8 cm in water) and heterogeneous phantom at lower point (26 cm physical depth, as shown in Fig. 1)

Description	Parameters		% Point dose differences				
	Field size (cm^2^)	Gantry angle	6 MV	10 MV	18 MV	6 MV FFF	10 MV FFF
Open field	10 × 10	0°	−0.4	−0.1	−0.9	−0.1	0.4
30° wedge	10 × 10	0°	−0.5	−0.6	0.6	NA	NA
110 cm SSD	20 × 20	0°	−1.2	−1.4	−0.8	0.2	0.3
Oblique field	30 × 30	20°	−1.2	−1.5	−1.8	−0.6	0.7
Rectangular field	20 × 5	0°	0.5	0.2	−0.3	0.4	0.7
Open field – lower point	10 × 10	0°	2.0	1.9	2.2	1.0	2.6

**Table 2:** Point dose differences in the 10 × 10 cm^2^ open field at 100 cm and 105 cm SSD in the Plastic water phantom. Note the depths of measurements are 1, 2, 2, and 3 cm for the 6, 9, 12, and 15 MeV electron energies, respectively

		% Point dose differences			
Description	Cone size	6 MeV	9 MeV	12 MeV	15 MeV
100 cm SSD	10 × 10 cm^2^	−0.3	0.2	−0.3	0.9
105 cm SSD	10 × 10 cm^2^	−1.5	0.7	1.7	0.9

**Table 3:** Comparison of density and mean HU values of CT electron density phantom inserts in Monaco TPS with the planning CT image

Insert	Relative electron density	Monaco density	CT HU	Monaco HU
Lung (LN‐300)	0.28	0.302	−665	−678
Lung (LN‐450)	0.4	0.413	−509	−524
Adipose (AP6)	0.9	0.922	−75	−69
Breast	0.96	0.957	−40	−33
Solid water	0.99	0.997	0	8
Liver (LV1)	1.07	1.07	94	83
Inner bone	1.09	1.087	190	203
Bone (B200)	1.11	1.091	202	224
Bone (CB2‐30% mineral)	1.28	1.238	443	427
Bone (CB2‐50% mineral)	1.47	1.413	756	748
Cortical bone (SB3)	1.69	1.605	1083	1105

### 3.C IMRT test fields

**Figure 3:**
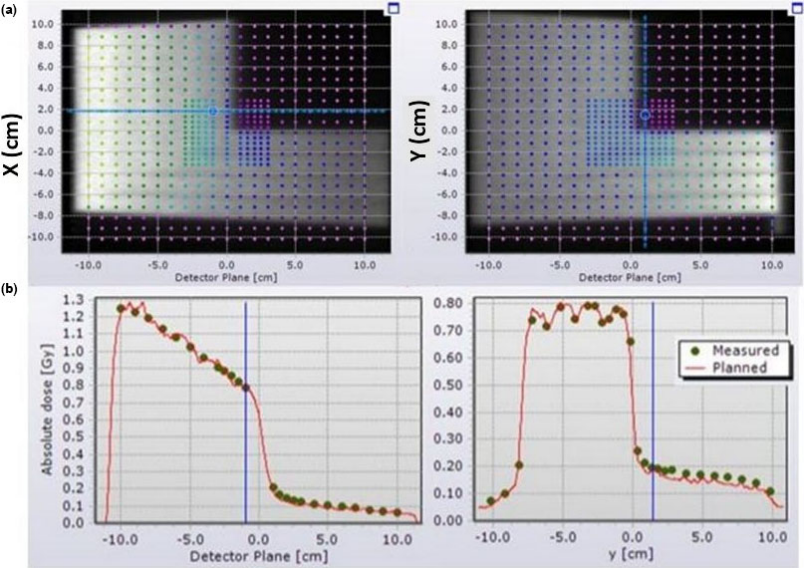
Beam profiles along the two detector plan in Delta^4^ of field “FourL” measured in a 6 MV photon beam. (a) The brightness level of the grayscale image indicates the planned dose distribution while the overlayed points show the dose measurements. (b) Inline and crossline profile of the measured and planned dose distribution.

The Monaco commissioning test fields yielded passing percentage of 93.8 ± 4.7% in the gamma analysis using 2% DD, 2 mm DTA criteria (as tabulated in Table 4). Among the eight test fields measured, the profile of the measured and planned FourL field, which had the lowest mean gamma passing rate of 87.6 ± 6.6%, is shown in Fig. 3.

**Table 4:** Parameters of the IMRT test fields and gamma passing rates based on 2% DD, 2 mm DTA criteria for the 5 photon energies

Fields	Description	Field size (cm^2^)	6 MV	10 MV	18 MV	6 MV FFF	10 MV FFF
10 × 10	Absolute dose calibration	10 × 10	99.3	93.5	91.5	97.6	91.3
20 × 20	Flatness, symmetry	20 × 20	96.4	94.4	93.0	96.0	97.6
3ABUT	3 abutted segments	6 × 24	98.9	89.5	93.0	92.0	95.5
DMLC	MLC offset	2 × 20	96.3	89.3	89.1	90.1	91.4
HIMRT	IMRT performance	Variable	99.7	94.3	94.2	99.9	94.8
HDMLC	DMLC performance	Variable	99.9	94.1	97.0	99.9	99.3
7SegA	Picket fence	2 × 24	96.2	85.4	91.6	97.1	96.3
FourL	4 L‐shaped MLC segments	Variable	93.6	76.9	85.8	91.4	90.3

### 3.D Patient data validation

Monaco TPS calculated dose distribution was compared against Delta^4^ measurements on two brain, two H&N, two lung, and two pelvis tumor sites. The mean, maximum, and minimum of gamma passing rates (3% DD, 3 mm DTA) based on the five measurements is tabulated in Table 5 along with the number of VMAT arcs. The percentage of points passing the gamma criteria, averaged across the sites, was 95.0 ± 0.9. Using a TG‐119‐based methodology, confidence limit is the summation of mean number of points failing the gamma criteria and 1.96 times the overall standard deviation. This gives a value of 6.7% and the expected gamma passing rate of 93.3% based on the 95% confidence limits. In the measurements, the number of plans with passing percent higher than expected gamma pass rates of 93.3% was 90%.

**Table 5:** The treatment sites, # arcs, and statistics on percentage of points passing gamma criteria of 3%/3 mm

Site	# Arcs	Mean	SD	Maximum	Minimum
Brain1	1	94.5	1.3	96.3	93.0
Brain2	2	93.7	1.3	95.4	91.9
H&N1	2	95.7	0.1	95.8	95.6
H&N2	2	96.8	0.5	97.5	96.4
Lung1	2	96.2	0.7	96.7	95.1
Lung2	2	93.48	0.8	94.4	92.5
Pelvis1	2	94.86	0.8	95.7	93.7
Pelvis2	2	94.84	1.1	96.2	93.8

## 4 DISCUSSION

MC simulation of radiation transport is one of the most accurate methods for predicting absorbed dose distributions which would help in estimation of clinical effectiveness. A major advantage of MC dose engine over conventional engines is the lower systematic error in dose computation.^1^ Verification of its dosimetric accuracy provides clinicians with a better understanding of dose in regions of heterogeneity, enabling more accurate treatment in regions which may have previously been complicated by systematic error in delivered dose. In addition, an advantage of MC calculation is prediction of dose in places where experimental measurements are impractical or less precise due to lack of electron equilibrium.^18^ The statistical uncertainty in MC‐based dose engine decreases with increasing number of iterations, albeit at the cost of computation time.

Monaco TPS has made MC dose computation of 3D through VMAT plans more accessible for clinics. With Monaco, the commissioning process is substantially different for physicists used to dose calculation engines based on modeling the energy fluence in house. The commissioning beam data are uploaded to a server (in this case, Elekta Physics Platform) where a model is created by Elekta. The physicist's job is then to verify the model with measurements of test fields and patient plans, as recommended in AAPM's MPPG report 5a.^16^ The reference condition dose and relative dose measurements are within the 0.5% and 2% tolerance recommendations of MPPG 5a (refer Table 3), respectively. The heterogeneity point dose measurement falls within the tolerance of 5%. The penumbra values of profile scans were within the 3‐mm tolerance mentioned in Table 5 in MPPG 5a. The reference and relative dose measurements for electron beams at two SSDs stated in Table 2 are within the 2% tolerance stated in MPPG report 5a.

AAPM Therapy Emerging Technology Assessment Work Group report on FFF beams mentions that unlike Varian, Elekta linacs have an independent energy set for FFF mode compared to the flattened counterparts that allows penetrative quality to match with the nominal value for that energy.^19^ PDDs of FFF beam show deeper *d*_max_ and steeper fall‐off with depth than the corresponding flattened energies. The collimator scatter factor and output factors were considerably lower for FFF beams for field sizes above 10 × 10 cm^2^ than their flattened counterparts.^20^ Kragl et al specifies removal of flattening filter softens the energy spectra and alters various dosimetric properties including scatter factor, surface dose, and leaf transmission.^21^ The readers are referred to Thompson et al for a detailed study on MLC characteristics in the Agility head.^9^ A systematic end‐to‐end testing to ensure confidence in modeling the dosimetric characteristics of the upgraded Elekta Agility head and FFF beams was addressed by Saenz et al.^22^

In all the patient IMRT QA validations, the average percentage of points passing gamma criteria (3% DD/3 mm DTA) exceeded 90%. Combining all the results of the eight site‐specific plans gives an overall average of 95% with a standard deviation of 0.9%. The confidence limit for these results was 6.7%, indicating that the percentage of points passing gamma criteria should be more than 93.3% approximately 95% of the time. From these collective measurements, 90% of the tests fell within the confidence limit. However, this analysis suffers from any statistical test dealing with limited number of dataset (n = 8) and no major changes in our clinic's dosimetry practice was required as a result of using a MC‐based planning system.

## 5 CONCLUSIONS

Point dose measurement agreed within 2% in a homogeneous phantom for all photon, electron beams and within 3% in a heterogeneous phantom for all photon beams. Monaco TPS commissioning was successfully verified on patient plans using dosimetric measurements with overall average gamma passing rates (3%/3 mm criteria) of 93.3% with 6.7% confidence limits.

## CONFLICT OF INTEREST

The authors declare no conflict of interest.
